# Multiple Robotic-Assisted Systems Versus Conventional Total Knee Arthroplasty: A Comparison of Two-Year Clinical Outcomes

**DOI:** 10.7759/cureus.105795

**Published:** 2026-03-24

**Authors:** Beruk Sherif, Gregorio Baek, Ji Won Lee, Christopher Jaicks, Thomas S Peacock, Jingyi Shao, Henry R Boucher

**Affiliations:** 1 Orthopedics, Georgetown University School of Medicine, Washington, DC, USA; 2 Orthopedics, MedStar Union Memorial Hospital, Baltimore, USA; 3 Orthopedic/Sports Medicine Research, MedStar Health Research Institute, Baltimore, USA

**Keywords:** outcome comparison, patient-reported outcome measures, postoperative complication, robotic-assisted total knee arthroplasty, robotic knee surgery, robotic total knee arthroplasty, total hip and knee arthroplasty, total knee arthroplasty technique, total knee arthroplasty (tka)

## Abstract

Background: While robotic-assisted (RA) total knee arthroplasty (TKA) offers improved precision in bone resection and implant alignment, disagreement exists regarding its clinical benefit compared to conventional TKA (conv-TKA), particularly concerning patient-reported outcome measures (PROMs). Multiple RA-TKA systems are in use, yet few studies compare outcomes among these systems. We aimed to report differences in clinical outcomes among two RA-TKA systems (i.e., Mako®, ROSA®) and conv-TKA.

Methods: This retrospective cohort study included TKAs performed by a single fellowship-trained orthopedic surgeon from January 1, 2016, to April 14, 2023. The electronic health record was reviewed for patient demographics and postoperative complications. PROMs (Short Form Health Survey (SF-12) and Oxford Knee Score (OKS)) were collected preoperatively and postoperatively (six months, one year, and two years) and analyzed using adjusted pairwise t-tests. Complication and revision rates were summarized using descriptive statistics.

Results: A total of 167 primary TKAs (n = 74 Mako, n = 29 ROSA, n = 64 conv-TKA) were included. At the six-month follow-up, the ROSA-TKA cohort had a significantly lower OKS than the conv-TKA cohort (-4.38, p = 0.042). Similarly, the Mako-TKA cohort had a significantly higher OKS than the ROSA-TKA cohort (4.79, p = 0.02). There were no significant differences in PROMs at the remaining follow-ups or in other pairwise comparisons. There was no statistically significant difference among groups in 90-day complications or TKA revisions.

Conclusions: Surgeons can achieve comparable clinical outcomes using various RA-TKA systems or conventional implants, without increased complications or revisions, while leveraging the enhanced implant placement accuracy of RA-TKA.

## Introduction

Robotic-assisted (RA) technology has gained popularity in total knee arthroplasty (TKA) for its enhanced precision in bone resection and implant alignment while minimizing tissue injury [1]. Using advanced technology, with or without imaging, RA systems generate a three-dimensional anatomical representation to optimize surgical planning. However, this technology has several drawbacks, such as high equipment costs, longer operative times, and associated learning curves, making its widespread adoption challenging [2]. A study on learning curves in RA-TKA reported a learning curve of seven cases, initially characterized by longer operative times than conventional TKA (conv-TKA) and increased anxiety levels among the surgical team [3]. Regardless of these disadvantages, increased patient demand and enhanced mechanical precision continue to make RA-TKA an attractive option among surgeons and patients [4].

Despite the increasing popularity of RA-TKA, its impact on clinical benefits compared with conv-TKA remains debated. Many studies utilize patient-reported outcome measures (PROMs) that assess functional status, health-related quality of life, pain, and symptom burden from the patient’s perspective to evaluate the perceived benefits of TKA [5]. Some studies of RA-TKA demonstrate improved short-term PROMs, higher patient satisfaction, faster recovery, shorter length of stay (LOS), and decreased revision rates compared to conv-TKA [6-9]. However, other studies have shown conflicting results, reporting either no difference in PROMs [10,11] or even improved PROMs with conv-TKA [12], making the benefits of RA-TKA versus conv-TKA in terms of PROMs inconclusive. With multiple RA-TKA systems available, each differing in cost, preoperative protocols, surgical techniques, and implant compatibility, comparative research is essential to determine which systems offer the greatest benefit in PROMs [13].

While there is a growing body of research on RA-TKA, there are several evidence gaps. Firstly, many studies generalize outcomes without differentiating between robotic systems [14,15]. A recent comparative study between Mako® Total Knee (Stryker, Kalamazoo, Michigan) and ROSA® Knee System (Zimmer Biomet, Warsaw, Indiana) found no differences in alignment accuracy or clinical outcomes at one-year follow-up [16]. However, this study lacks a control group, such as conv-TKA, which can provide an established benchmark. Since various RA-TKA platforms may have system-dependent PROM benefits, comparative studies will allow for a more nuanced understanding of their differences [13]. Secondly, while a few studies comparing RA-TKA versus conv-TKA have adjusted for patient comorbidities [17-20], which have a significant impact on recovery after TKA [21], many studies have not controlled for this important variable. Thirdly, there is conflicting literature examining the effect of RA-TKA on postoperative complications. Meta-analyses assessing surgery-related complications found no significant difference between RA-TKA and conv-TKA [22,23]. However, these studies primarily analyzed older robotic systems (i.e., ROBODOC (THINK Surgical Inc., Fremont, California) and NAVIO (Smith & Nephew, Memphis, Tennessee) and others not widely used in the United States (i.e., CASPAR (Universal Robot Systems, Rastatt, Germany), HURWA (BEIJING HURWA-ROBOT Technology Co. Ltd), and Yuanhua (Yuanhua Orthopedic Robotics, China)), although one study included Mako [23]. Other large database studies have reported lower complication rates with RA-TKA than conv-TKA, though they did not specify the robotic systems utilized [24,25].

Given the limited comparative-effectiveness studies on PROMs across different RA-TKA systems, the few studies that adjust for preoperative comorbidities [17-20,25], and the ongoing debate regarding complication rates, there remains a need for research utilizing PROMs as a primary metric of TKA comparison. To address this gap, we aimed to compare PROMs and complication rates at a two-year follow-up among patients who underwent TKA with two different robotic systems (i.e., Mako, ROSA) or conv-TKA [26].

This work has been previously presented in part as posters and oral presentations at multiple scientific meetings, including the MedStar Georgetown Summer Research Capstone (Washington, DC; October 18, 2023), the Medical Student Orthopedic Society Symposium (virtual; April 21, 2024), the George M. Kober Student Research Day (virtual; April 26, 2024), and the Virginia Orthopedic Society 77th Annual Meeting (Tysons, Virginia; May 3, 2024).

## Materials and methods

Ethical review and study design

This was a single-site retrospective study involving a single arthroplasty practice of a fellowship-trained joint replacement surgeon. The surgeon is certified in robotic-assisted TKA systems and has employed these systems since 2017. The study was approved as an exempt chart review by the MedStar Health Research Institute (IRB ID: STUDY00007436).

Data source

Patient demographic and surgical data were collected from the electronic health records (EHR) system. Patient PROM data were collected from the Outcomes Based Electronic Research Database (OBERD).

Patient selection

Patients were eligible if they were at least 18 years old at the time of surgery, underwent RA-TKA or conv-TKA by the principal investigator between January 1, 2016, and April 14, 2023, and had available preoperative PROMs. Exclusion criteria included revision procedures, absence of preoperative PROMs, or missing postoperative PROMs at six months, one year, or two years. Among the 322 consecutive cases that underwent Mako-TKA within the study period, 74 were included and 248 were excluded. Among the 315 consecutive cases that underwent ROSA-TKA, 29 were included and 286 were excluded. Among the 138 consecutive cases that underwent conv-TKA, 64 were included and 74 were excluded. There was a high number of exclusions due to incomplete preoperative and postoperative PROMs.

Data collection

Patient Demographics

Data parameters collected from chart review included age, sex, body mass index (BMI), date of surgery, implant type (Mako, ROSA, or conventional), preoperative comorbidities, 90-day postoperative complications, and revisions. Preoperative comorbidities were assessed using the Elixhauser Comorbidity Index (ECI) [27]. The 90-day postoperative complications included periprosthetic infections, deep vein thrombosis (DVT), pulmonary embolism (PE), arthrofibrosis requiring manipulation under anesthesia (MUA), and periprosthetic fracture.

Patient-Reported Outcome Measures

PROMs utilized in this study included the Short Form Health Survey (SF-12), Oxford Knee Score (OKS), and Knee Injury and Osteoarthritis Outcome Score-Joint Replacement (KOOS-JR). Scores were obtained from the OBERD database preoperatively and at six months, one year, and two years postoperatively.

The SF-12 survey comprises physical and mental components, with higher scores indicating greater physical and mental quality of life [28]. It evaluates activity level, functional accomplishment, pain, and mental state. The SF-12 score ranges from 0 to 100, with a mean of 50 equating to the average health status of the general population. It is a reliable and valid instrument to assess health-related quality of life [28].

The OKS survey evaluates pain and physical function of the knee [29]. The score ranges from 0 to 48, with higher scores indicating increased knee function and decreased pain. The OKS survey has been shown to have a high degree of reproducibility, construct validity, and sensitivity to clinically significant changes in outcome measures for patients undergoing TKA [29].

The KOOS-JR score incorporates pain, symptoms, and functional limitations of the knee into a single score representing overall knee function and health [30]. As a shortened version of the Knee Injury and Osteoarthritis Outcome Score (KOOS), a validated PROM widely used and adopted by the Centers for Medicare and Medicaid Services (CMS) [31], the KOOS-JR score has been shown to accurately incorporate aspects of knee pain, overall symptoms, and limitations in activities of daily living (ADLs) that are directly pertinent to patients with knee osteoarthritis [30]. This score ranges from 0 to 100, with higher scores representing increased knee function and health.

Surgical technique

Prior to the introduction of RA-TKA in the principal investigator’s practice, they used either a cemented conventional implant or a commercially available custom-designed cemented implant. For TKAs performed with Mako or ROSA robotic assistance, the corresponding manufacturer’s cemented and cementless implants were used.

Fixation strategy (cemented versus cementless) was tailored to patient factors such as age, bone quality, and activity level. In general, cementless implants were preferentially used in younger, higher-demand patients with good bone stock, whereas cemented implants were used in patients with poorer bone quality, lower activity levels, or intraoperative concern for suboptimal press-fit fixation. For the RA-TKA cohorts, all knees utilized non-posterior-stabilized implant designs, as described below. The surgeon used a kinematic alignment strategy for all RA-TKAs. Conventional TKAs were performed using standard instrumentation, as described below.

The conv-TKA cohort consisted of either the Columbus® Cruciate-Retaining (CR; Aesculap, B. Braun, Tuttlingen, Germany) implant or the custom-designed Conformis iTotal® CR (Billerica, Massachusetts) implant. For the non-custom implant, an intramedullary guide was used to resect the femur with an intended alignment of five degrees of valgus. The remainder of the femoral cuts were made with a four-in-one guide based on the posterior medial femoral condyle, with rotation determined from the anterior-posterior femoral axis. An extramedullary guide was used to make the tibial cut with a goal of neutral alignment and three degrees of posterior slope. Spacer blocks were used to balance the extension and flexion gaps, with ligament release as necessary. For the custom implant, personalized cutting guides were used for the bone resections, and spacer blocks were used to assess ligament balance, with releases performed as needed. The knee components were cemented in both custom and non-custom implants.

The Mako® Total Knee System (Stryker, Mahwah, New Jersey) was one of the RA-TKA systems in this study. It is a computed tomography (CT)-based, semi-active robotic arm that provides haptic guidance for the saw. Before surgery, all patients scheduled for Mako-TKA underwent a lower extremity CT scan for anatomical assessment and three-dimensional (3D) surgical planning. The Mako system technician and the surgeon collaboratively set the preoperative plan. The surgery began with exposure of the knee joint and placement of trackers and reference pins through stab wounds in the tibia and femur. The anatomy, range of motion, alignment, and ligament tension of the knee were then registered and applied to the preoperative CT-based 3D model. The implants were virtually manipulated for size, alignment, balance, bone resection depth, gap data, and integrated limb alignment. Once the surgical plan was finalized, the Mako robotic arm was used to perform the femoral and tibial cuts under haptic guidance. Trial implants were placed, and the navigation system was used to check the range of motion, alignment, and balance. Once these metrics were satisfactory, the trial implants, trackers, and reference pins were removed before placement of the final implant. The final implant utilized was either the press-fit or cemented Stryker Triathlon Condylar Stabilizing® (Stryker, Mahwah, New Jersey).

The ROSA® Knee System (Zimmer Biomet, Warsaw, Indiana) was the second RA-TKA system in this study. This system did not require a preoperative CT for surgical planning. Intraoperatively, the knee joint was exposed via a standard medial parapatellar approach. Next, trackers were placed through stab incisions in the tibia and femur. The anatomical landmarks of the knee were registered in the ROSA system to create a 3D model of the knee in space, followed by registration of the range of motion and gap balance. Once the surgical plan was finalized, the ROSA robotic arm was used to place guides for femoral and tibial cuts. Trial implants were placed, and navigation confirmed a satisfactory range of motion, alignment, and balance before removal of the trial implants, trackers, and reference pins. The final implant, a cemented or cementless Zimmer Biomet Persona Medial Congruent Bearing® (Zimmer Biomet, Warsaw, Indiana), was placed.

The preoperative, anesthetic, intraoperative, and postoperative protocols were consistent across the three cohorts. All surgeries were performed through a medial parapatellar approach under tourniquet control. Implant selection, posterior cruciate ligament (PCL) management, and patellar resurfacing followed the surgeon’s long-standing practice: the PCL was either retained or pie-crusted for proper anterior-posterior tracking, and the patella was selectively resurfaced based on the presence of significant cartilage loss in the patellofemoral compartment. A drain was placed and removed within 24 hours postoperatively. Postoperative care included weight-bearing as tolerated, multimodal oral pain management, DVT prophylaxis, and physical therapy starting on the day of surgery, followed by a rehabilitation protocol focused on early range of motion, gait training with assistive devices as needed, mobility, and progressive strengthening.

Patients were followed up in the clinic at 4 and 10 weeks postoperatively. They were also instructed to follow up at one and two years postoperatively. PROM surveys for SF-12, OKS, and KOOS-JR were sent electronically at six-month, one-year, and two-year postoperative follow-ups. The OBERD system automatically collected completed surveys as scores in its database. Patients with missing PROMs within the appropriate follow-up window were sent additional reminders to complete their surveys.

Statistical analysis

We performed an a priori power analysis based on a previous study [32], which determined that a sample size of 28 in each cohort would achieve 81% power to detect a minimum clinically important difference (MCID) of seven points for OKS and SF-12. Accounting for a 20% loss to follow-up in each group, we used a conservative sample size of 34 in each group, increasing the total required sample size to 102.

Most patients in this study used the OKS to assess functional knee outcomes. More recent participants, however, utilized both the OKS and KOOS-JR, with some of the most recent exclusively using KOOS-JR, partially due to the anticipated CMS adoption mentioned above [31]. Subsequently, some patients had OKS but lacked KOOS-JR scores at certain time points (e.g., preoperative and 6 months), while having KOOS-JR data at later follow-ups (e.g., one year and two years). For patients with only KOOS-JR scores available at a given follow-up, we used a validated crosswalk table to convert KOOS-JR scores to corresponding OKS scores [33]. KOOS-JR scores that did not have exact conversions to OKS scores were rounded to the nearest KOOS-JR score with an OKS conversion based on the crosswalk table [33]. The crosswalk table was used to convert a total of 36 KOOS-JR scores (2 Mako, 34 ROSA), distributed across follow-up time points (7 preoperative, 10 at six months, 12 at one year, and 7 at two years).

The missingness rate was less than 55% for the Mako-TKA cohort, less than 80% for the ROSA-TKA cohort, and less than 45% at all postoperative time points for the conv-TKA cohort. Missing postoperative PROM data were considered likely consistent with a missing at random (MAR) mechanism, as the missingness at one- and two-year follow-ups for the ROSA group was likely attributable to the later introduction of this platform into the surgeon’s practice in 2021. Consequently, we performed a complete-case analysis, and only patients with complete PROM datasets were included.

We used descriptive statistics to compare the baseline characteristics of the Mako-TKA, ROSA-TKA, and conv-TKA groups. Continuous variables were summarized as means and standard deviations. Categorical/binary variables were represented as counts and proportions. Analysis of variance (ANOVA) was used for normally distributed continuous variables, and the Kruskal-Wallis rank sum test was used for non-normally distributed continuous variables (i.e., ECI). ECI scores were reported as a composite score. Descriptive statistics were also used to represent the incidence of 90-day postoperative complications and revisions in the three groups. Pairwise t-tests of estimated marginal means derived from a mixed-effects model adjusting for age, sex, BMI, and ECI were used to compare PROMs from each postoperative time point (six-month, one-year, and two-year) to preoperative PROMs to assess improvements within and among the three groups. A p-value of 0.05 was used to determine statistical significance, with adjustments made using the Tukey method where appropriate. The software R, Version 4.4.1, was used to perform all statistical analyses.

Manuscript editing

Artificial intelligence (AI) tools, specifically ChatGPT (OpenAI, San Francisco, California), were used to assist with language editing and improve this manuscript's clarity and flow. The AI tool was not used for data analysis, scientific interpretation, or content generation. The authors independently developed and verified all scientific content and interpretations to ensure accuracy and integrity.

## Results

A total of 167 knees were included for analysis (n = 74 Mako, n = 29 ROSA, n = 64 conv-TKA). Mean age was 66.15 ± 7.30 years, and 54.5% were female, with a mean BMI of 32.58 ± 6.32 kg/m^2^. Mean ECI was 2.56 ± 1.73 (Table 1).

**Table 1:** Patient demographics P-values for age and BMI were generated using ANOVA, and p-values for ECI were generated using the Kruskal–Wallis rank sum test. Sex was reported descriptively without statistical comparison. BMI, body mass index; ECI, Elixhauser Comorbidity Index.

Patient Characteristic	MAKO, N = 74	ROSA, N = 29	Conventional, N = 64	P-value
Age (years), mean (SD)	66.7 (6.7)	66.4 (7.4)	65.4 (8.0)	0.422
Sex, n (%)				0.108
Female	34 (45.9)	16 (55.2)	41 (64.1)	
Male	40 (54.1)	13 (44.8)	23 (35.9)	
BMI (kg/m^2^), mean (SD)	32.9 (6.5)	30.6 (5.9)	33.1 (6.4)	0.126
ECI, mean (SD)	2.8 (1.7)	2.4 (1.7)	2.6 (1.8)	0.476

**Figure 1:**
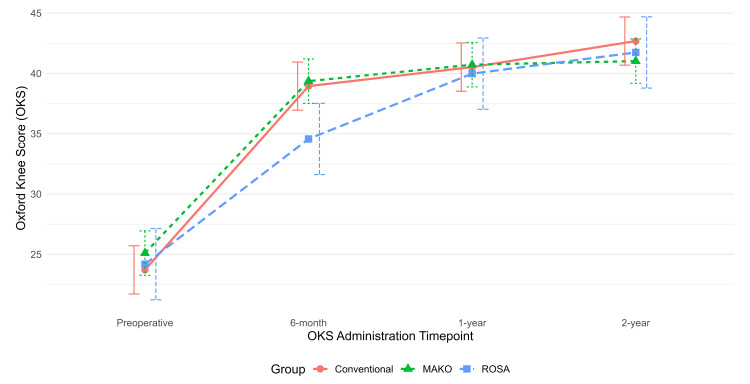
Time-series plot of Oxford Knee Score (OKS) at six-month, one-year, and two-year follow-ups, with 95% confidence intervals shown as error bars

**Figure 2:**
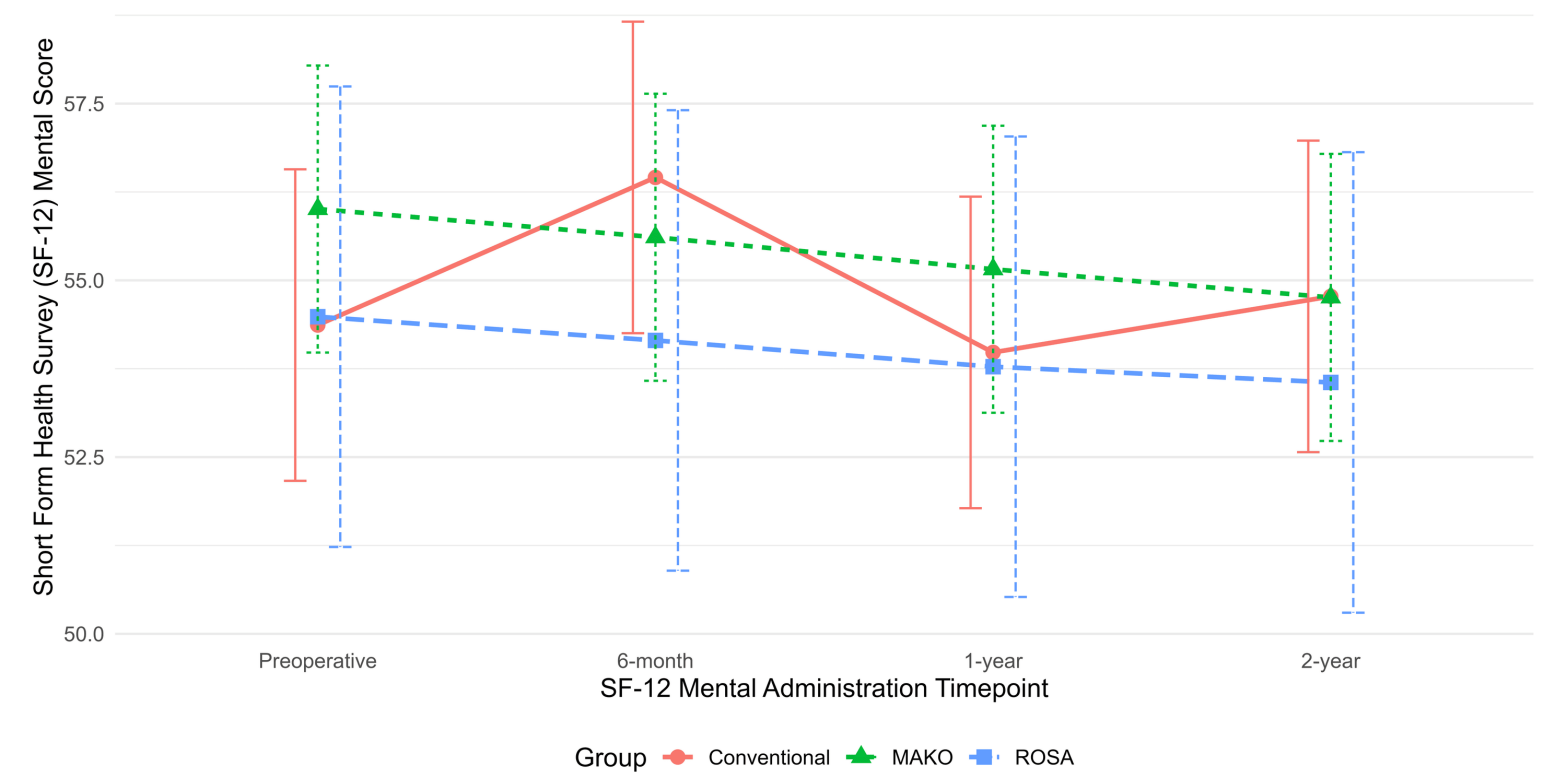
Time-series plot of Short Form Health Survey (SF-12) mental scores at six-month, one-year, and two-year follow-ups, with 95% confidence intervals shown as error bars

**Figure 3:**
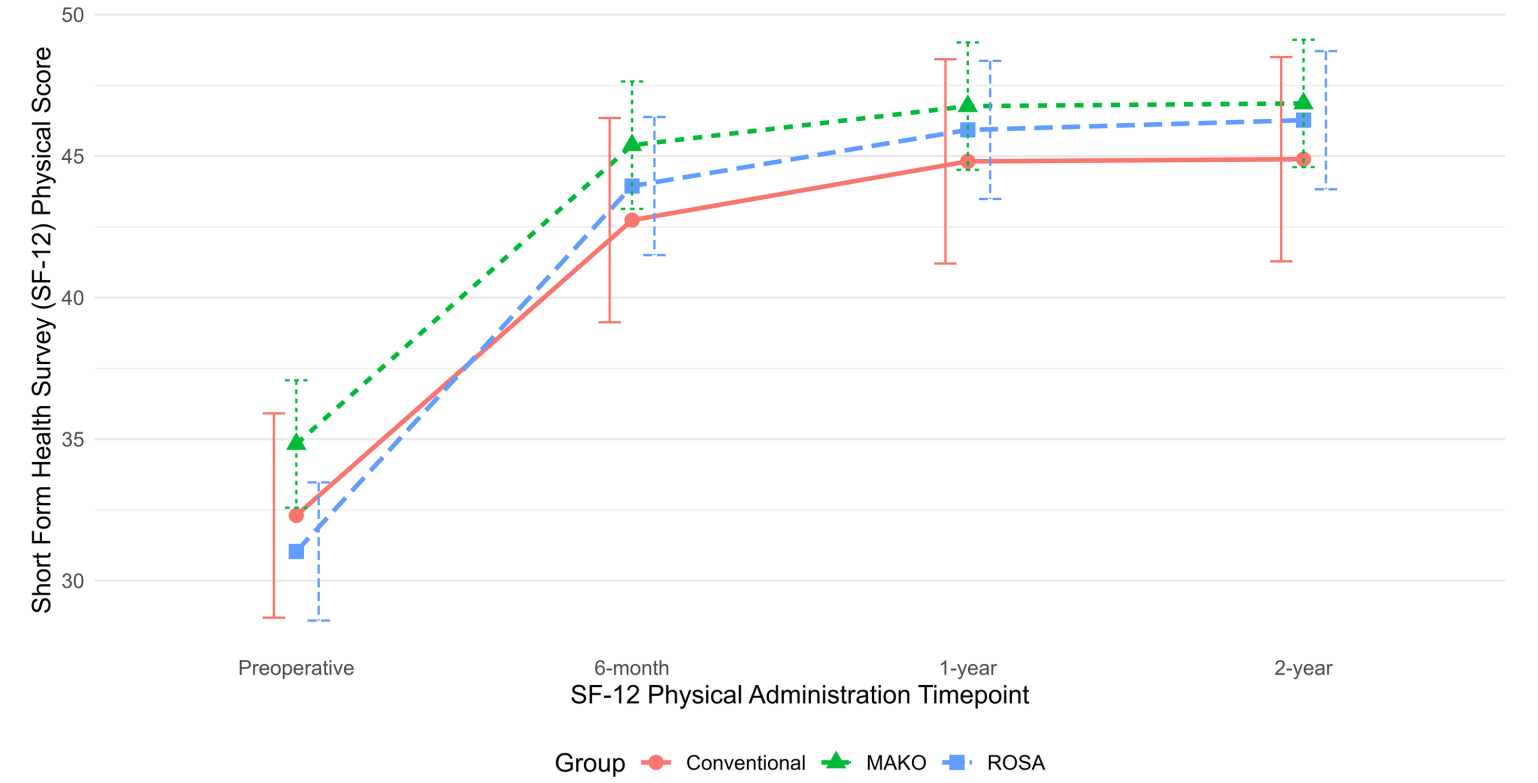
Time-series plot of Short Form Health Survey (SF-12) physical scores at six-month, one-year, and two-year follow-ups, with 95% confidence intervals shown as error bars

Preoperative and postoperative PROMs at six-month, one-year, and two-year follow-up were analyzed with adjustment for age, sex, BMI, and ECI (Table 2). Longitudinal trends in OKS, SF-12 Mental, and SF-12 Physical scores are displayed in corresponding time-series plots (Figures 1-3).

**Table 2:** Preoperative and postoperative PROMs at six-month, one-year, and two-year follow-ups adjusted for age, sex, BMI, and ECI CI, confidence interval; OKS, Oxford Knee Score; SF-12, Short Form Health Survey; ECI, Elixhauser Comorbidity Index; PROM, patient-reported outcome measure.

Outcome Measure	MAKO (95% CI), N=74	ROSA (95% CI), N=29	Conventional (95% CI), N=64
OKS			
Preoperative	25.1 (23.3, 26.9)	24.2 (21.2, 27.1)	23.7 (21.7, 25.7)
6-month	39.4 (37.5, 41.2)	34.6 (31.6, 37.5)	38.9 (36.9, 40.9)
1-year	40.7 (38.9, 42.6)	40 (37, 42.9)	40.5 (38.5, 42.5)
2-year	41 (39.2, 42.9)	41.7 (38.8, 44.7)	42.7 (40.7, 44.7)
SF-12 mental			
Preoperative	56 (54, 58)	54.5 (51.2, 57.7)	54.4 (52.2, 56.6)
6-month	55.6 (53.6, 57.6)	54.2 (50.9, 57.4)	56.5 (54.3, 58.7)
1-year	55.2 (53.1, 57.2)	53.8 (50.5, 57)	54 (51.8, 56.2)
2-year	54.8 (52.7, 56.8)	53.6 (50.3, 56.8)	54.8 (52.6, 57)
SF-12 physical			
Preoperative	34.8 (32.6, 37.1)	32.3 (28.7, 35.9)	31 (28.6, 33.5)
6-month	45.4 (43.1, 47.6)	42.7 (39.1, 46.3)	43.9 (41.5, 46.4)
1-year	46.8 (44.5, 49)	44.8 (41.2, 48.4)	45.9 (43.5, 48.4)
2-year	46.9 (44.6, 49.1)	44.9 (41.3, 48.5)	46.3 (43.8, 48.7)

The adjusted pairwise comparisons of PROMs among the three different TKA implants at each time point (preoperative, six-month, one-year, and two-year) are illustrated in Table 3. At the six-month follow-up, the ROSA cohort had a significantly lower OKS than the conv-TKA cohort (-4.38, 95% CI: -8.64, -0.12; p=0.042). Additionally, the Mako cohort had a significantly higher OKS than the ROSA cohort (4.79, 95% CI: 0.61, 8.97; p=0.02).

**Table 3:** Preoperative and postoperative PROMs pairwise comparison between three implants adjusted for age, sex, BMI, and ECI *Statistical significance at p < 0.05. P-values for postoperative PROM pairwise comparisons were generated using ANOVA with Tukey post hoc adjustment. CI, confidence interval; OKS, Oxford Knee Score; SF-12, Short Form Health Survey.

Outcome Measure	MAKO - Conventional (95% CI)	P-value	ROSA - Conventional (95% CI)	P-value	MAKO - ROSA (95% CI)	P-value
OKS						
Preoperative	1.39 (-1.87, 4.66)	0.576	0.48 (-3.79, 4.74)	0.963	0.91 (-3.26, 5.09)	0.864
6-month	0.41 (-2.85, 3.68)	0.952	-4.38 (-8.64, -0.12)	0.042*	4.79 (0.61, 8.97)	0.02*
1-year	0.2 (-3.07, 3.46)	0.989	-0.54 (-4.81, 3.72)	0.952	0.74 (-3.44, 4.92)	0.908
2-year	-1.66 (-4.92, 1.61)	0.456	-0.94 (-5.2, 3.32)	0.862	-0.72 (-4.9, 3.46)	0.914
SF-12 mental						
Preoperative	1.64 (-1.96, 5.24)	0.531	0.12 (-4.58, 4.82)	0.998	1.52 (-3.08, 6.13)	0.716
6-month	-0.85 (-4.45, 2.75)	0.845	-2.31 (-7, 2.39)	0.481	1.46 (-3.15, 6.07)	0.736
1-year	1.18 (-2.42, 4.78)	0.722	-0.2 (-4.9, 4.5)	0.994	1.38 (-3.23, 5.99)	0.761
2-year	-0.01 (-3.62, 3.59)	1	-1.22 (-5.92, 3.48)	0.815	1.2 (-3.4, 5.81)	0.812
SF-12 physical						
Preoperative	3.8 (-0.19, 7.78)	0.066	1.27 (-3.93, 6.48)	0.834	2.52 (-2.58, 7.63)	0.476
6-month	1.44 (-2.54, 5.43)	0.671	-1.2 (-6.41, 4)	0.849	2.65 (-2.46, 7.75)	0.441
1-year	0.84 (-3.15, 4.83)	0.874	-1.11 (-6.32, 4.09)	0.87	1.95 (-3.15, 7.05)	0.641
2-year	0.59 (-3.4, 4.58)	0.935	-1.38 (-6.58, 3.83)	0.808	1.97 (-3.13, 7.07)	0.635

There were no statistically significant differences in SF-12 (physical or mental) in pairwise comparisons among the three cohorts at any time point. No pairwise comparisons of adjusted PROMs between the TKA implants exceeded the MCID threshold (7 for OKS and SF-12 physical) to be considered clinically significant.

The adjusted pairwise comparisons of longitudinal within-group differences in postoperative versus preoperative PROMs among the three TKA implant groups are illustrated in Table 4. There were no statistically significant differences in the degree of improvement among the three cohorts for any postoperative PROMs compared with preoperative PROMs.

**Table 4:** Difference in PROMs between the three implants comparing different postoperative time points to preoperative adjusted for age, sex, BMI, and ECI P-values were generated using pairwise t-tests of estimated marginal means derived from a mixed-effects model, with adjustments made via the Tukey method. CI, confidence interval; OKS, Oxford Knee Score; SF-12, Short Form Health Survey; PROM, patient-reported outcome measure; ECI, Elixhauser Comorbidity Index.

Outcome Measure	MAKO - Conventional (95% CI)	P-value	ROSA - Conventional (95% CI)	P-value	MAKO - ROSA (95% CI)	P-value
OKS						
6 months - Preoperative	-0.98 (-4.88, 2.92)	0.980	-4.86 (-9.97, 0.26)	0.072	3.88 (-1.13, 8.88)	0.215
1 year - Preoperative	-1.19 (-5.09, 2.71)	0.950	-1.02 (-6.13, 4.10)	0.994	-0.17 (-5.18, 4.83)	1
2-year - Preoperative	-3.05 (-6.95, 0.85)	0.207	-1.42 (-6.5, 3.70)	0.968	-1.63 (-6.64, 3.37)	0.933
SF-12 mental						
6 months - Preoperative	-2.49 (-6.18, 1.20)	0.357	-2.42 (-7.26, 2.41)	0.675	-0.06 (-4.80, 4.67)	1
1 year - Preoperative	-0.46 (-4.15, 3.22)	1	-0.32 (-5.16, 4.52)	1	-0.14 (-4.88, 4.59)	1
2 years - Preoperative	-1.66 (-5.34, 2.03)	0.769	-1.34 (-6.17, 3.5)	0.969	-0.32 (-5.05, 4.41)	1
SF-12 physical						
6 months - Preoperative	-2.35 (-6.92, 2.22)	0.650	-2.48 (-8.47, 3.52)	0.827	0.12 (-5.74, 5.99)	1
1 year - Preoperative	-2.96 (-7.53, 1.61)	0.403	-2.38 (-8.48, 3.61)	0.850	-0.57 (-6.44, 5.29)	1
2 years - Preoperative	-3.21 (-7.78, 1.37)	0.315	-2.65 (-8.65, 3.34)	0.781	-0.55 (-6.42, 5.31)	1

The complication and revision rates of the three cohorts are depicted in Table 5. There was no statistically significant difference in the overall 90-day complication rates (5.4% Mako, 3.4% ROSA, 1.6% conv-TKA; p = 0.454) among groups. None of the included patients developed a postoperative infection, PE, or periprosthetic fracture. Four Mako patients developed postoperative complications within three months: DVT (n = 1) and arthrofibrosis requiring MUA (n = 3). One ROSA patient developed a postoperative complication within three months: arthrofibrosis requiring MUA (n = 1). One conv-TKA patient developed a postoperative complication within three months: DVT (n = 1). There was no statistically significant difference in revision rates (4.1% Mako, 3.4% ROSA, 0% conv-TKA; p = 0.234) among the three groups. Three Mako patients required revision surgeries within two years postoperatively: knee instability (n = 1), arthrofibrosis with pain after MUA (n = 1), and patellar component loosening (n = 1). One ROSA patient required revision surgery within two years postoperatively for press-fit tibial component loosening (n = 1).

**Table 5:** Complication and revision rates P-values for complication and revision rates were generated using Fisher’s exact test. DVT, deep vein thrombosis; PE, pulmonary embolism; MUA, requiring manipulation under anesthesia.

Clinical Outcome	MAKO, N = 74	ROSA, N = 29	Conventional, N = 64	P-value
Complications				0.454
Infection, n (%)	0	0	0	
DVT, n (%)	1 (1.4)	0	1 (1.6)	
PE, n (%)	0	0	0	
Arthrofibrosis MUA, n (%)	3 (4.1)	1 (3.4)	0	
Periprosthetic fracture, n (%)	0	0	0	
Revision, n (%)	3 (4.1)	1 (3.4)	0	0.234

## Discussion

We found a statistically significant difference in the OKS score between the ROSA and conv-TKA cohorts and between the Mako and ROSA cohorts at the six-month follow-up. However, these differences did not meet the previously mentioned MCID of 7 based on our power analysis. There were no significant differences in SF-12 pairwise comparisons among the three cohorts at any postoperative time points. No significant differences in the degree of improvement in postoperative PROMs compared to preoperative values were observed among the three cohorts at any postoperative time points. No significant differences in 90-day complication rates or revisions were observed among groups.

To our knowledge, this is the first study to provide comparative data on PROMs among multiple robotic systems and conventional TKA at six-month, one-year, and two-year follow-ups. The goal of comparative effectiveness research is to explain the benefits and harms of various interventions used to treat a clinical condition [34]. PROMs provide unfiltered information directly from the patient’s perspective to evaluate TKA-related pain and function [26]. The surveys used were validated [34] and can therefore effectively assess comparative effectiveness among various treatment interventions such as Mako-TKA, ROSA-TKA, and conv-TKA.

Our findings support previous studies that reported no differences and contrast with studies that demonstrated significant differences in postoperative PROMs among RA-TKA systems and conv-TKA. The transiently lower six-month OKS observed in the ROSA cohort should be interpreted cautiously, as this group had the smallest sample size, the highest proportion of missing PROM data, and included early platform adoption cases during the COVID-19 period, as further discussed below. Zhou et al. found no significant difference in postoperative Western Ontario and McMaster University Osteoarthritis Index (WOMAC) or Knee Society Score (KSS) between Mako and ROSA-TKA at the one-year follow-up [16]. Similarly, long-term comparison studies between RA-TKA and conv-TKA with a minimum 10-year follow-up using WOMAC, KSS, and SF-12 found no significant differences [10,11]. Conversely, recent short-term studies have shown significant differences in postoperative PROMs for RA-TKA compared to conv-TKA. While a recent meta-analysis found greater improvements in KSS and WOMAC scores with conv-TKA at a minimum six-month follow-up [12], another study observed significantly better two-year WOMAC scores in patients undergoing Mako-TKA compared to conv-TKA [35]. Another study using the Short Form Health Survey (SF-36) to compare outcomes found that RA-TKA patients experienced significantly improved role and vitality emotional measures at two-year follow-up; however, no differences were found in OKS or KSS [36].

Our study found no significant differences in complication rates, contrasting with previous literature suggesting that RA-TKA may be associated with lower complication rates than conv-TKA [20,24,25]. Previous studies have shown decreased complication rates and LOS in RA-TKA compared with conv-TKA [24,25]. These large database studies reported lower rates of PE in RA-TKA: 0.1% versus 0.2% (p < 0.05) [24], and 0.074% versus 0.228% (p < 0.0001) [25]. Both studies also reported significantly lower rates of DVT in RA-TKA: 0.1% versus 0.2% (p < 0.05) [24], and 0.187% versus 0.327% (p = 0.010) [25]. Our findings likely differ due to the smaller sample size and low incidence of complications. We found no significant differences in revision rates among groups, aligning with the existing literature [20]. A study utilizing the American Joint Replacement Registry to compare revision rates between RA-TKA and conv-TKA found no difference in revision rate or the odds of revision within two years [20].

Limitations and future perspectives

There are multiple limitations to this study. Firstly, this study is limited by its nature as a single-surgeon series and may not be generalizable to all arthroplasty surgeons, who may have different training and experience. Secondly, we excluded patients with incomplete preoperative or postoperative PROM surveys, which may have affected our findings. The number of patients excluded markedly decreased the sample size for analysis. Thirdly, there is a risk of selection bias related to both survey completion and treatment choice. While PROMs provide valuable information on treatment effectiveness, barriers to recovery, and overall perception of improvement from the patient’s perspective, their reliability depends on patient compliance with self-reporting, which may be compromised due to various patient-related factors [5]. Previous studies have found that a decreased likelihood of survey completion was significantly associated with lower income, government-sponsored insurance, Black race, opioid use, lower expectations, and lower physical activity [37]. Future studies may identify populations at high risk for noncompliance with surveys and implement targeted strategies to enhance engagement. This may involve educating patients on the significance of PROMs in treatment evaluation and decision-making while addressing barriers hindering participation (e.g., digital barriers) [5]. Additionally, as RA systems were adopted into the surgeon’s practice, patients earlier in this study were given a choice between RA and conv-TKA. Although there were no differences in demographics among the cohorts of patients undergoing RA-TKA or conv-TKA, unmeasured confounders in patient characteristics may have influenced the selection of RA-TKA as opposed to conv-TKA.

The surgeon completed training and certification courses from both RA-TKA system manufacturers before use, and early adoption cases for both RA-TKA cohorts were included in the study. Prior studies suggest that PROMs and complication rates may not differ significantly during the RA-TKA learning curve [38]; however, we note this as a potential source of variability because early adoption cases for both robotic platforms were included in our analysis. Additionally, the highest proportion of missing data was observed in the ROSA cohort, mostly due to the shorter follow-up period. As previously mentioned, the ROSA system was introduced later (2021) in the study period, meaning many patients had not yet reached their two-year follow-up at the time of analysis, limiting our sample size and making this group more susceptible to selection and attrition bias. Early ROSA cases also coincided with the COVID-19 pandemic, during which rehabilitation access and follow-up patterns were atypical, which may partially explain the transiently lower six-month OKS [39]. The lower six-month OKS observed in the ROSA cohort did not exceed the MCID threshold and did not persist at the one- or two-year follow-up.

With regard to statistical analysis, the need to convert some KOOS-JR scores to OKS using the crosswalk is another limitation that may have introduced measurement imprecision. Although the crosswalk enabled inclusion of patients who otherwise would have had incomplete outcome data, this limitation also highlights the need for more comprehensive PROM crosswalk tools with continuous values as clinical practices evolve. The Forgotten Joint Score (FJS) is a commonly reported PROM tool in arthroplasty outcomes research [40]; however, the senior surgeon’s practice did not routinely collect FJS during the study period. Incorporating FJS in future prospective studies may provide additional insight into joint awareness and patient satisfaction.

With respect to surgical methods, there was heterogeneity in fixation method (cemented versus cementless), implant manufacturer, constraint design, and utilization of patellar resurfacing. These factors were tailored to individual patient characteristics and reflected the evolution of the surgeon’s long-standing clinical practice, but they were not systematically recorded during initial data collection and could not be reliably reconstructed or adjusted for after dataset de-identification. Accordingly, these unmeasured variables may have acted as potential confounders influencing PROMs and early recovery. Prior literature has generally shown comparable PROMs between cemented and cementless TKA [41], no significant two-year PROM differences among CR, anterior-stabilized (AS), and posterior-stabilized (PS) implant designs [42], and conflicting evidence regarding the effect of patellar resurfacing on PROMs, with many studies reporting equivalent outcomes between resurfaced and non-resurfaced patellae [43].

Despite these limitations, the study’s strengths include direct comparative analysis of multiple RA-TKA systems and conv-TKA as a control group. We used validated PROMs to assess clinical outcomes, adjusted for comorbidities, analyzed complications and revisions, and provided findings for patients with a two-year follow-up.

## Conclusions

We observed a statistically significant difference in the OKS score among groups at the six-month follow-up. We did not observe differences in PROMs (OKS and SF-12), 90-day complications, or revisions at any other postoperative follow-up among Mako, ROSA, and conv-TKA. These findings suggest that surgeons can achieve comparable clinical outcomes with multiple RA systems and conventional TKA, without increased complications or revisions, while potentially benefiting from the enhanced implant placement accuracy associated with RA-TKA in prior studies; however, alignment was not directly evaluated in the present cohort. It is also important to recognize and address the high rates of PROM noncompliance in certain patient populations, emphasizing the need for improvement to generate more representative data on treatment effectiveness and patient outcomes among patients undergoing knee replacement surgery. Moreover, further long-term comparative studies of system-specific RA-TKA outcomes utilizing PROMs are warranted.
